# Human Lymphadenopathy Caused by Ratborne *Bartonella*, Tbilisi, Georgia

**DOI:** 10.3201/eid2203.151823

**Published:** 2016-03

**Authors:** George Kandelaki, Lile Malania, Ying Bai, Neli Chakvetadze, Guram Katsitadze, Paata Imnadze, Christina Nelson, Shimon Harrus, Michael Kosoy

**Affiliations:** Central University Hospital, Tbilisi (G. Kandelaki);; National Center for Disease Control & Public Health, Tbilisi, Georgia (G. Kandelaki, L. Malania, N. Chakvetadze, G. Katsitadze, P. Imnadze);; Centers for Disease Control and Prevention, Fort Collins, Colorado; USA (Y. Bai, C. Nelson, M. Kosoy);; Hebrew University of Jerusalem, Rehovot, Israel (S. Harrus)

**Keywords:** Bartonella, cat scratch disease, rats, Tbilisi, Georgia, vector-borne infections, zoonoses, bacteria, lymphadenopathy

## Abstract

Lymphadenopathy and fever that developed in a woman in Tbilisi, Georgia, most likely were caused by a ratborne *Bartonella* strain related *B. tribocorum* and *B. elizabethae*. The finding suggests that this *Bartonella* strain could be spread by infected rats and represents a potential human risk.

Cat scratch disease caused by *Bartonella henselae* is a major cause of unilateral reginal lymphadenitis in children and adults (*1*). We report a case of lymphadenopathy and fever in a woman in Tbilisi, Georgia, that most likely was caused by a ratborne *Bartonella* strain.

## The Study

 In 2012, an 18-year-old woman with no major medical history sought care at an outpatient infectious diseases clinic in Tbilisi with a 2-week history of weakness, malaise, fever >38°C for the previous 10 days, enlarging right neck mass, and occasional night sweats. She lived in a residential building in an urban area within Tbilisi. She denied recent travel outside the city, contacts with sick persons, exposure to farm animals, or having pets at home. Physical examination indicated right cervical lymphadenopathy with multiple enlarged, soft, tender lymph nodes, 1 of which was fluctuant on palpation. Ultrasound showed 4 enlarged lymph nodes: 2 in the anterior cervical region (14 mm and 17 mm) and 2 in the posterior cervical region (29 and 38 mm). The largest lymph node had central attenuation with a hypoechoic area suggestive of pus. Laboratory test results were as follows: leukocyte count 9.2 cells/μL (reference 4.0–11.0 cells/μL) with 6.7% (reference 2.5%–7.5%) neutrophils; platelets 358,000/μL (reference 150,000–450,000/μL); hemoglobin 15.4 g/dL (reference 14.0–17.5 g/dL); C-reactive protein 16 mg/L (reference 0–10 mg/L); and erythrocyte sedimentation rate 56 mm/h (reference <30 mm/h). Chest radiograph showed no abnormalities. Serum was negative for antibodies against cytomegalovirus, Epstein-Barr virus, *Toxoplasma*, and HIV. Tuberculin skin test result of 4-mm induration was considered negative. 

Ultrasound-guided aspiration of the largest lymph node yielded 2 mL of cloudy yellow fluid. Gram stain, acid-fast stain, bacterial culture, and fungal culture of the aspirate were all negative. Histopathologic examination demonstrated a nonspecific inflammatory response without evidence of granulomas or malignant cells.

Cat scratch disease was presumptively diagnosed on the basis of lymphadenopathy and clinical characteristics, and *B*. *henselae* was suspected as the etiologic agent, although the patient denied any contact with cats. The lymph node aspirate was submitted to the National Center for Disease Control & Public Health (Tbilisi) for molecular diagnostic testing for *Bartonella*. Genomic DNA was extracted from the lymph node aspirate by using a QIAamp tissue kit (QIAGEN, Valencia, CA, USA) and was analyzed by using conventional PCR targeting a 338-bp fragment of the *glt*A gene (*1*), a molecular target routinely used for detecting *Bartonella* DNA. The test resulted in amplification of the specific target, which suggested a potential *Bartonella* species. Before the PCR result was available, the patient was empirically prescribed amoxicillin/clavulanic acid treatment. After receiving the PCR results suggesting *Bartonella* DNA in the aspirate sample, the drug regimen was switched to azithromycin 500 mg every 8 hours on the first day, then 250 mg every 8 hours daily for 4 additional days. Fever resolved in 2 weeks, and lymphadenopathy gradually improved during the next 4–5 weeks. Weakness and malaise resolved within 2 months.

The DNA was forwarded to the Bartonella and Rodent-Borne Diseases Laboratory of the US Centers for Disease Control and Prevention’s Division of Vector-Borne Diseases (Fort Collins, CO, USA) for further characterization. Seven targets (*glt*A, *nuo*G, *rib*C, *rpo*B, *fts*Z, *ssr*A, and internal transcribed spacer [ITS]), all which have been previously used for *Bartonella* descriptions (*2*), were amplified. All positive PCR products were purified by using QIAquick PCR Purification Kit (QIAGEN) and sequenced in both directions by using an Applied Biosystems Model 3130 Genetic Analyzer (Applied Biosystems, Foster City, CA, USA). The sequences obtained were aligned by each locus and compared with all known *Bartonella* species by using the ClustalW program within DNASTAR Lasergene package (DNASTAR, Madison, WI, USA). The neighbor-joining method by Kimura 2-parameter distance method was used (1,000 bootstrap replicates).

Sequence analyses of all 7 molecular targets demonstrated that the bacterial DNA belongs to a *Bartonella* species within the *B. elizabethae* species complex, which represents an assemblage of species and strains associated with rats of the genus *Rattus* (*3*). By individual locus, the DNA was closer to *B. tribocorum* than to any other *Bartonella* species by 2 markers (*glt*A [96.1%] and *rpo*B [100%]) and was closer to *B. elizabethae* by all other markers (*nuo*G [99.9%], *rib*C [99.2%], *fts*Z [98.5%], and *ssr*A [99.4%]) and by ITS (99.4%). Comparison of concatenated sequences of all 7 loci indicated the identified genotype had a divergence of 3.4% with *B. elizabethae* and 5.6% with *B. tribocorum*. Additional sequence queries resulted in identification of the Tel Aviv (TA) strain of *Bartonella*, which was prevalent and the only identified strain among black rats (*Rattus rattus*) captured in Tel Aviv, Israel (*4*). For any of the 4 markers used in both studies (*rib*C, *rpo*B, *glt*A, and ITS), the genotype identified in the patient was indistinguishable from the TA strain. The *glt*A sequence from the patient’s aspirate also was indistinguishable by the *glt*A from *Bartonella* genotypes identified in a rat from Porto Santo Island, Portugal (*5*), and in 4 rats from Dhaka, Bangladesh (*6*).

## Conclusions

The invasion of rats into urban ecosystems and their establishment in such areas can have major implications for human health (*7*). *Bartonella* species and genotypes detected in *Rattus* rats are clustered into a defined phylogenetic lineage that can be subdivided into several subclusters (*3*,*6*). *B. elizabethae* and related species of *Bartonella* have not been detected in rodent hosts except for rats of genera *Rattus* and *Bandicota* (*3*,*6*,*7*). A recent genetic analysis of *Bartonella* strains obtained from rats from 17 countries demonstrated that this bacterial complex evolved and diversified in Southeast Asia before being disseminated by *R. rattus* and *R. norvegicus* to other parts of the globe (*8*).

*B. elizabethae* was first isolated from a US patient with endocarditis in 1993 (*9*) and subsequently was found in rats from many countries (*2*,*4*). Investigation of febrile human patients from Thailand demonstrated that 8 of the 14 *Bartonella* genotypes identified in patients were similar or identical to homologous sequences identified in rats and were closely related to *B. elizabethae*, *B. rattimassiliensis*, or *B. tribocorum* (*10*).

The identification of bacteria that share genes specific for rat-associated *Bartonella* species in a lymph node aspirate suggests that the finding could be associated with commensal rats occupying residential areas of Tbilisi. The patient did not recall rats in the building but had noticed them in waste containers outside the building. The most striking finding was the identity of this genotype with TA strain. Of 21 *Bartonella* isolates cultured from blood from 62 commensal rats captured in Tel Aviv, 10 isolates were genetically characterized by 6 markers, and all the isolates were identical to each other and closely related to both *B. tribocorum* and *B. elizabethae* (*3*). Identification of the identical strains in urban rats from Portugal and Bangladesh suggests much wider distribution of this strain.

The clinical picture for the patient we report was typical for clinical manifestations of cat scratch disease, which is commonly caused by *B. henselae* (*11*). Nevertheless, evidence is increasing that rodentborne *Bartonella* species can cause diverse clinical signs and symptoms, including fever, myocarditis, endocarditis, neuroretinitis, and lymphadenitis (*12*). *Bartonella* species between rodents appear to be transmitted mainly by fleas (*13*). The Oriental rat flea (*Xenopsylla cheopis*) commonly infests commensal rats within cities and can readily bite humans without being noticed. The detection of a rat-associated *Bartonella* species in the capital of Georgia raises public health concerns and highlights the need to further explore its zoonotic potential and pathogenic characteristics.
